# Correction: *Ab initio* study of the electronic states of V_3_Si in momentum space

**DOI:** 10.1039/d3ra90090h

**Published:** 2023-09-22

**Authors:** Saloni Sharma, Nikhil Joshi, Vijay Maurya, K. B. Joshi

**Affiliations:** a Department of Physics, M L Sukhadia University Udaipur-313001 India cmsmlsu@gmail.com

## Abstract

Correction for ‘*Ab initio* study of the electronic states of V_3_Si in momentum space’ by Saloni Sharma *et al.*, *RSC Adv.*, 2023, **13**, 25836–25845, https://doi.org/10.1039/D3RA04535H.

The authors regret that there was an error in eqn (7). The correct equation is as shown below:
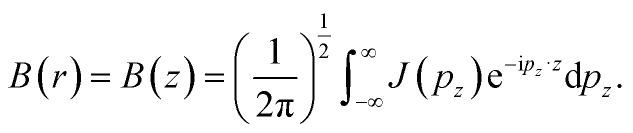


The Royal Society of Chemistry apologises for these errors and any consequent inconvenience to authors and readers.

## Supplementary Material

